# Near-Drowning: To Be or Not to Be … Is It the Question?

**DOI:** 10.3390/ijerph15040760

**Published:** 2018-04-15

**Authors:** Bruno Mégarbane, Hossein Mehdaoui, Dabor Résière

**Affiliations:** 1 Department of Medical and Toxicological Critical Care, Lariboisière Hospital, Paris-Diderot University, 75010 Paris, France; 2 Critical Care Unit, University Hospital of Martinique, 97200 Martinique, French West Indies; Hossein.MEHDAOUI@chu-martinique.fr (H.M.); Dabor.RESIERE@chu-martinique.fr (D.R.)

We thank Dr. Queiroga and collaborators for the very nice letter [1] advising us to definitively abandon the term of “near drowning”, based on the Utstein-style guidelines for the uniform reporting of data from drowning, which was published in 2003 [2]. Interestingly, a 2005 systematic review of drowning reports found twenty different definitions for drowning, thirteen different definitions for near-drowning, and thirteen related terms in the reviewed articles [3]. Near-drowning, as explained in the guidelines from the American Heart Association, usually refers to a critical aquatic predicament resolved by successful water rescue, definition implying certain recovery once the victim is removed from the water. However, like in our patients, this was not always the case; patients regaining consciousness after near-drowning had subsequently died due to aspiration pneumonia, even though no apparent clinical signs could be found on the initial physical examination. Therefore, an international group with scientific expertise in the field of drowning research that was supported by the European Resuscitation Council and the American Heart Association encouraged the abandonment of the ‘near-drowning’ term that was considered to generate confusion [4].

Surprisingly, these recommendations in contrast to all other international guidelines on cardiac arrest reporting remain poorly recognized by the majority of the non-specialized emergency physicians and intensivists. Of the 2010–2016 published articles, 32% included outdated and non-uniform drowning terminology and definitions, despite a 11%-decrease as compared with the 2003–2010 period, supporting the persistence of room for improvement [5]. At least 23 PubMed abstracts in the 2017–2018 period, additionally to ours, reported on “near-drowning” cases, including in journals of reference like Clinical Infectious Diseases [6]. Recently, StatPearls, which is a free academic e-learning management system that is intended for a broader medical readership even published an article that was dedicated to near-drowning, which was defined as nonfatal drowning, i.e., survival, at least temporarily, after suffocation by submersion in a liquid medium, including or not loss of consciousness [7]. All of the persistent inconsistencies and the absence of uniformity in the use of definitions across the drowning literature clearly suggest that a consensus is far to be reached. We thus strongly approve Dr. Byard’s conditions for a term to be useful, i.e., general acceptance, widespread and regular application, use in reputable peer-reviewed journals, and evidence to be of assistance in case management and analysis [8]. We thus should recognize that “near drowning” has served these purposes during many years, although it appears now to be black-listed by the consensus.
